# Modified enhanced recovery after surgery (ERAS) protocols for patients with obstructive colorectal cancer

**DOI:** 10.1186/s12893-017-0213-2

**Published:** 2017-02-16

**Authors:** Dai Shida, Kyoko Tagawa, Kentaro Inada, Keiichi Nasu, Yasuji Seyama, Tsuyoshi Maeshiro, Sachio Miyamoto, Satoru Inoue, Nobutaka Umekita

**Affiliations:** 1 Colorectal Surgery Division, National Cancer Center Hospital, 5-1-1 Tsukiji, Chuo-ku, Tokyo, 1040045 Japan; 2 Department of Anesthesiology, Tokyo Metropolitan Bokutoh Hospital, 4-23-15 Koto-bashi, Sumida-ku, Tokyo, 1308575 Japan; 3 Department of Surgery, Tokyo Metropolitan Bokutoh Hospital, 4-23-15 Koto-bashi, Sumida-ku, Tokyo, 1308575 Japan

**Keywords:** ERAS, Obstructive colorectal cancer, Length of hospital stay

## Abstract

**Background:**

Enhanced recovery after surgery (ERAS) protocols are now well-known to be useful for elective colorectal surgery, as they result in shorter hospital stays without adversely affecting morbidity. However, the efficacy and safety of ERAS protocols for patients with obstructive colorectal cancer have yet to be clarified.

**Methods:**

We evaluated 122 consecutive resections for obstructive colorectal cancer performed between July 2008 and November 2012 at Tokyo Metropolitan Bokutoh Hospital. Patients with rupture or impending rupture and those who received simple colostomy were excluded. The first set of 42 patients was treated based on traditional protocols, and the latter 80 according to modified ERAS protocols. The main endpoints were length of postoperative hospital stay, postoperative short-term morbidity, rate of readmission within 30 days, and mortality. Differences in modified ERAS protocols relative to traditional care include intensive preoperative counseling (by both surgeons and anesthesiologists), perioperative fluid management (avoidance of sodium/fluid overload), shortening of postoperative fasting period and early provision of oral nutrition, intraoperative warm air body heating, enforced postoperative mobilization, stimulation of gut motility, early removal of urinary catheter, and a multidisciplinary team approach to care.

**Results:**

Median (interquartile range) postoperative hospital stay was 10 (10–14.25) days in the traditional group, and seven (7–8.75) days in the ERAS group, showing a 3-day reduction in hospital stay (*p* < 0.01). According to the Clavien-Dindo classification, overall incidences of grade 2 or higher postoperative complications for the traditional and ERAS groups were 15 and 10% (*p* = 0.48), and 30-day readmission rates were 0 and 1.3% (*p* = 1.00), respectively. As for mortality, one patient in the traditional group died and none in the ERAS group (*p* = 0.34).

**Conclusion:**

Modified ERAS protocols for obstructive colorectal cancer reduced hospital stay without adversely affecting morbidity, indicating that ERAS protocols are feasible for patients with obstructive colorectal cancer.

## Background

Enhanced recovery after surgery (ERAS) protocols comprise a combination of various perioperative patient care methods using a multidisciplinary team approach that integrates evidence-based interventions that reduce surgical stress, maintain postoperative physiological function, and accelerate recovery in patients undergoing major surgery [1]. ERAS protocols involve pre-, intra-, and postoperative elements, and their fundamental aspects focus on preoperative counseling, no fasting, optimal fluid management, decreased use of tubes, opioid-sparing analgesia, and early mobilization [1]. ERAS protocols have been most extensively studied in the context of colorectal surgery, and recommendations regarding perioperative care in colorectal surgery from the ERAS society are being continuously updated as new information becomes available [2–4]. ERAS protocols are now well-known to be useful for elective colorectal surgery, as they result in shorter hospital stays without adversely affecting morbidity [5, 6].

Studies on ERAS protocols have mainly originated from European countries and the United States (where the term, ‘fast track surgery’, is also used), and only a few have been conducted in Asian countries [7]. ERAS protocols were introduced in Japan around 2008, and were initially introduced in our hospital to patients who underwent colorectal resection in July 2010 [8, 9]. We previously demonstrated that ERAS protocols for elective colorectal surgery helped reduce the length of postoperative hospital stay without adversely affecting morbidity, indicating that ERAS protocols are feasible and effective in Japan, with its unique medical culture and public health insurance system [8].

Although a large number of clinical studies have confirmed the benefits of ERAS protocols in elective surgery, their efficacy in the context of emergent surgery, such as obstructive colorectal cancer surgery, remains uncertain, given the significant challenges of applying ERAS protocols in the emergency setting. For example, patients with obstructive colorectal cancer, which is associated with a high rate of postoperative complications and prolonged hospital stays, cannot be prepared in the same way preoperatively and often differ from patients who undergo elective surgery. That is, obstructive colorectal cancer patients cannot eat orally before surgery and must fast preoperatively—this is in direct contradiction with ERAS protocols, which require no preoperative fasting [4]. However, many intra-operative and postoperative evidence-based ERAS elements, such as postoperative ‘no fasting’, can also be applied to emergent colectomy [10].

In the context of emergent colorectal surgery, recent reports from Thailand [11], Switzerland [10], and Australia [12] found that modified ERAS protocols are safe. Accordingly, we extended the application of modified ERAS protocols to patients with obstructive colorectal cancer. To this end, this study aimed to evaluate the efficacy and safety of ERAS protocols for patients with obstructive colorectal cancer.

## Methods

### Study population

A total of 122 consecutive patients undergoing colorectal resection for obstructive colorectal cancer between July 2008 and November 2012 at Tokyo Metropolitan Bokutoh Hospital, a standard Japanese general hospital, were included in the study. Exclusion criteria were patients with bowel perforation (*n* = 12) and patients who underwent stoma construction without bowel resection (*n* = 31). During the first 2 years of the study, patients were treated according to care routines considered traditional at that time in Japan (traditional group, *n* = 42). After specific ERAS protocols were introduced in our hospital in 2010 [8, 9], these protocols have become the standard of care for all patients undergoing elective colorectal resection. Since July 2010, we adopted ERAS protocols not only for patients who undergo elective colorectal surgery, but also for patients with obstructive colorectal cancer who undergo surgery. The second group of consecutive patients after July 2010 were treated with modified ERAS protocols (ERAS group, *n* = 80). The same colorectal surgeon primarily cared for patients during both study periods, and all procedures were performed by the same team of surgeons. The technical aspects of surgery, such as the choice of staplers and other instruments, and the choice of antibiotics, did not change during the study period. All patients received a one-stage operation, without stoma construction. This study was approved by the Tokyo Bokutoh Metropolitan Hospital institutional review board (IRB) (IRB code: 25 –Heisei23) and written informed consent was obtained from all patients.

### Perioperative protocols

We described regular ERAS protocols of Tokyo Metropolitan Bokutoh Hospital previously [8]. Intensive pre-admission counselling, no pre- and postoperative fasting (provision of oral nutrition), avoidance of sodium/fluid overload, intraoperative warm-air body heating, enforced postoperative mobilization, and multimodal team care were among the main changes brought about by the introduction of ERAS protocols [8]. Thus, regular ERAS protocols require no preoperative fasting. For patients with obstructive colorectal cancer, it is impossible to have a meal orally before surgery as well as just after surgery. Thus, we modified the ERAS protocols to tailor them to patients with obstructive colorectal cancer, with which the second group of consecutive 80 patients after July 2010 were treated (Table 1). Main differences of the modified ERAS protocols relative to traditional care for patients with obstructive colorectal cancer include the following: intensive preoperative counseling (by both surgeons and anesthesiologists), perioperative fluid management (avoidance of sodium/fluid overload), shortening postoperative fasting and the early provision of oral nutrition, intraoperative warm air body heating, enforced postoperative mobilization, stimulation of gut motility (use of oral magnesium oxide), early removal of urinary catheter, and a multidisciplinary team approach to care. Some elements of ERAS protocols, such as the use of thoracic epidural anesthesia/analgesia and avoidance of pre-anesthetic medication, were already routine practices at the initiation of the study, and consequently, were part of traditional perioperative care. Defined discharge criteria, such as tolerance of food, adequate pain control, independence in basic activities of daily living, and a willingness to go home, did not change throughout the study.

**Table 1:** Changes in perioperative care for patients with obstructive colorectal cancer

	Traditional care	Modified ERAS
preoperative counseling	advice given only by surgeons	intensive (by both surgeons and anesthesiologist)
preoperative fasting (oral intake)	no food and no drink	
preoperative bowel preparation	no	
perioperative fluid management (avoidance of sodium/fluid overload)	no	yes (goal-directed fluid therapy)
intraoperative warm air body heating	sometimes	always
nasogastric tube	used (remove by POD3)	used (remove on POD1)
postoperative fasting	no oral intake for 3 days after surgery	start drinking oral hydration solution by POD2
	start eating soup on POD5	start eating rice on POD3
routine postoperative mobilization care	yes (walk by POD2)	enforced (walk in the morning of POD1)
non-opiate oral analgesics/NSAIDs	no	given routinely
stimulation of gut motility	no	yes (use of oral magnesium oxide)
early removal of urinary catheter	no	Yes
multidisciplinary team approach	few cases	all cases
anesthesia and analgesic	combination epidural analgesia and general anesthesia (use of remifentanil)	
avoidance of pre-anesthetic medication (no premed)	Yes	
abstinence from smoking and drinking	Yes	

### Data collection

The following demographic and perioperative data were collected: gender, age, tumor location, emergent or elective operation, stage of colorectal cancer (based on AJCC TNM classification), length of postoperative hospital stay, and complications. Emergent surgery was considered surgery performed just after unplanned hospital admission. Elective surgeries included, for example, cases with preoperative decompression of dilated bowel by transanal drainage tube [13] and nasogastric tube. Complications were defined as grade 2 or higher complications within 30 days of surgery, according to the Clavien-Dindo classification. The number of dissected lymph nodes confirmed by pathologists was also recorded as an indicator of the quality of surgery [14].

### Statistical analysis

Demographic and perioperative data are presented as median (interquartile range), box and whisker plot (25^th^, 75^th^ percentiles), mean ± SD, or number (%), as appropriate. Unless otherwise stated, comparisons are between traditional and ERAS groups. Statistical evaluations were performed using two-way analysis of variance. Wilcoxon signed-rank test was used to assess continuous outcomes, and Fisher’s exact test for binary outcomes. All statistical analyses were performed using the JMP12 software program (SAS Institute Japan Ltd., Tokyo, Japan). *P* < 0.05 was considered statistically significant.

## Results

A total of 122 consecutive patients with obstructive colorectal cancer were enrolled. The traditional group included 42 patients, and the modified ERAS group included 80 patients. Patient characteristics are shown in Table 2. The two groups were statistically similar with respect to gender (*p* = 0.551) and age (*p* = 0.825). Ratios of emergent surgery were also similar (*p* = 0.345). Most cases of elective surgeons received preoperative decompression of dilated bowel by transanal drainage tube or nasogastric tube. No patient had a colonic stent positioned in a bridge to surgery policy, because Japanese health insurance did not cover the treatment of colonic stent at that time in Japan. Tumor location and stage distribution were almost similar between the two groups. The types of surgeries slightly differed between two groups (*p* = 0.021). Laparoscopic surgery (*n* = 4) was only performed in the ERAS group. In addition, the numbers of dissected lymph nodes were 33.0 ± 17.3 in the traditional group and 38.1 ± 21.4 in the ERAS group, with no significant difference (*p* = 0.264). These results suggest that sufficient lymph node dissection was performed in both groups.

**Table 2:** Patient demographics and characteristics

	Traditional	ERAS	
	2008.07-2010.06	2010.07-2012.11	p
	*n* = 42	*n* = 80	
Gender (male/female)	25/17	52/28	0.551
Age (years)	67.5 (41–88)	69 (39–92)	0.825
Location			
right-side colon	17 (41%)	27 (34%)	
left-side colon	19 (45%)	40 (50%)	
rectum	6 (14%)	13 (16%)	0.762
Surgery			
emergent	22 (52%)	49 (61%)	
elective (transanal drainage, etc.)	20 (48%)	31 (39%)	0.345
Types of surgery			
Open- colectomy	33 (78%)	48 (60%)	
Open- low anterior resection	7 (17%)	23 (29%)	
Open- abdominoperineal resection	2 (5%)	0	
Open- total/subtotal colectomy	0	5 (6%)	
Laparoscopic colectomy	0	4 (5%)	0.021
Number of retrieved lymph nodes	33.0 ± 17.3	38.1 ± 21.4	0.264
Stage			
I	1 (2%)	0	
II	10 (24%)	32 (40%)	
III	18 (43%)	20 (25%)	
IV	13 (31%)	28 (35%)	0.073

**Fig. 1:**
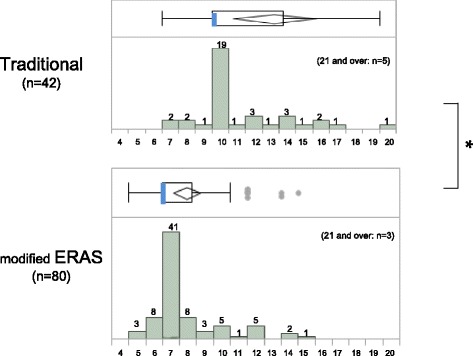
Postoperative length of hospital stay in the traditional group and modified ERAS group. Postoperative length of hospital stay is presented as a histogram and by box and whisker plots (25^th^, 75^th^ percentiles) for both the traditional group and modified ERAS group. Median LOS is indicated with blue thick vertical bars. Vertical boundaries of the boxes represent the first and third quartiles. Rhombuses indicate means. *p < 0.05

Outcomes with regard to postoperative hospital stay are shown in Fig. 1. The median (interquartile range) postoperative hospital stay was 10 (10–14.25) days in the traditional group, and seven (7–8.75) days in the ERAS group, i.e., a 3-day reduction in hospital stay (*p* < 0.05). There was a dramatic increase in the proportion of patients who were discharged within 1 week, which increased from 5% in the traditional group to 65% in the ERAS group.

Postoperative outcomes are summarized in Table 3. Overall incidences of grade 2 or higher postoperative complications were 15 and 10% for the traditional and ERAS groups (*p* = 0.48), respectively. There were no significant differences in the rates of anastomotic leakage, postoperative ileus, pneumonia, and overall complications. There were no deaths in the modified ERAS group; one patient in the traditional group died during the postoperative course due to exacerbation of aspiration pneumonia due to preoperative vomiting. Rates of 30-day readmission were 2.5% in the traditional group and 0% in the ERAS group, with no significant difference between the two groups. There was also no significant difference in 30-day reoperation rates between the ERAS and traditional groups (2.5 and 0%, respectively).

**Table 3:** Postoperative outcomes

	Traditional (*n* = 42)	ERAS (*n* = 80)	*p* value
Postoperative complications ^a^	6 (15%)	8 (10%)	0.480
anastomotic leakage	0 (0%)	2 (2.5%)	0.545
ileus	2 (5%)	3 (3.8%)	1.000
pneumonia	2 (5%)	0 (0%)	0.122
others	2 (5%)	3 (3.8%)	1.000
Readmission within 30 days	0 (0%)	1 (1.3%)	1.000
Reoperation within 30 days	1 (2.5%)	0 (0%)	0.344
Mortality	1 (2.5%)	0 (0%)	0.344

^a^ According to Clavien-Dindo classification (grade 2 or higher)

## Discussion

Although many elements of ERAS protocols can be equally applied to emergent and elective settings, no study has assessed whether ERAS protocols might benefit patients with obstructive colorectal cancer. The present study demonstrated that modified ERAS protocols for obstructive colorectal cancer can successfully accelerate patient recovery without increasing postoperative morbidity or readmission rates, and importantly, without compromising patient safety. These results suggest that ERAS protocols are also feasible for patients with obstructive colorectal cancer.

Similar to our present conclusions, several studies have recently reported that ERAS protocols, although there were several small differences from ours, can be safely applied to the setting of emergent colorectal surgery [10–12]. Another study (a randomized controlled clinical trial) demonstrated the feasibility of ERAS protocols in emergent surgery for perforated peptic ulcer disease [15], with primary endpoints of length of hospital stay, morbidity, and mortality.

Some of the ERAS elements, such as intensive preoperative counseling, perioperative fluid management, and enforced postoperative mobilization, are obviously feasible in obstructive cancer. In the present study, modified ERAS protocols shortened the median length of hospital stay by 3 days. The magnitude of reduction in hospital stay is fairly comparable to those reported in studies of ERAS protocols used in elective colorectal surgery [8]. The reduction in hospital stay resulting from modified ERAS protocols is likely attributed to a combination of multimodal perioperative interventions (rather than any single element) that aimed to attenuate the metabolic response to surgery, to support the recovery of organ function, and to preserve the postoperative immune system [11]. A multidisciplinary team approach by surgeons, anesthesiologists, nurses, physiotherapist, and nutritionists, is indispensable.

Whereas most studies investigating the effectiveness of ERAS protocols include doctor-reported outcomes such as length of hospital stay, postoperative complications, and mortality, patient-reported outcomes also need to be assessed. Using the 40-item quality of recovery score (QoR-40), a recovery-specific and patient-rated questionnaire, we recently reported that QoR-40 scores dropped significantly on postoperative days 1 and 3, but dramatically recovered up to baseline on postoperative day 6 [9]. This suggests that the quality of recovery, as indicated by patient-reported outcomes, is in agreement with decisions to discharge patients with colorectal cancer treated under an ERAS protocol at around postoperative day 6 [9]. With respect to patients of the present study who were treated with modified ERAS protocols, seven patients answered the QoR-40 questionnaire, with results similar to the aforementioned study, i.e., scores decreased on postoperative days 1 and 3, but dramatically recovered up to baseline on postoperative day 6 (data not shown). For patients with obstructive colorectal cancer being treated under ERAS protocols, the quality of recovery was in agreement with discharge around postoperative day 6, a result similar to that of patients who undergo elective colorectal surgery.

Some limitation of this study included a relatively small sample size with a selected group of patients. Low risk patients were likely to be included, while high-risk patients with obstructive colorectal cancer or locally far advanced cancer patients were subjected to less invasive management such as diverting colostomy. Another limitation of this study was that the study was not a randomized controlled trial but a retrospective study. However, we believe the study has its place. When a new approach is being introduced, it is important to gather worldwide results since regional differences do occur. This study serves as a pilot study for future prospective and preferably randomized studies in the region, which may add to the growing evidence and a final evaluation of the approach. It is recognized that this patient group is difficult to include in a largescale randomized setup, and the retrospective approach in the presented study seems warranted to explore the hypothesis.

## Conclusions

In summary, the present study found that modified ERAS protocols for patients with obstructive colorectal cancer reduce hospital stay without adversely affecting morbidity. These results indicate that ERAS is feasible and efficient not only for patients who undergo elective colorectal cancer surgery, but also for those who undergo obstructive colorectal cancer surgery.

## Abbreviations

ERAS: Enhanced recovery after surgery
IRB: Institutional review board
LOS: Length of hospital stay
POD: Postoperative day
